# Neonatal supplementation of oleamide during suckling ameliorates maternal postpartum sleep interruption-induced neural impairment and endocannabinoid dysfunction in early adolescent offspring rats

**DOI:** 10.3389/fnut.2025.1566182

**Published:** 2025-05-12

**Authors:** Linxi Qian, Tao Zheng, Bowen Zhao, Weiye Wang, Yifan Wu

**Affiliations:** ^1^Shanghai Key Laboratory of Pediatric Gastroenterology and Nutrition, Xinhua Hospital, Shanghai Jiao Tong University, School of Medicine, Shanghai, China; ^2^Shanghai Institute for Pediatric Research, Xinhua Hospital, Shanghai Jiao Tong University, School of Medicine, Shanghai, China; ^3^Department of Obstetrics, Xinhua Hospital, Shanghai Jiao Tong University, School of Medicine, Shanghai, China; ^4^Department of Clinical Nutrition, College of Health Science and Technology, Shanghai Jiao Tong University School of Medicine, Shanghai, China; ^5^Ministry of Education and Shanghai Key Laboratory of Children’s Environmental Health, Xinhua Hospital, Shanghai Jiao Tong University, School of Medicine, Shanghai, China

**Keywords:** oleamide, maternal sleep interruption, breast milk, cognitive function, endocannabinoids

## Abstract

**Introduction:**

Postpartum sleep disturbances in women are common and can significantly affect maternal mental health and breastfeeding. However, the impact of sleep disruptions in lactating mothers on the neurological and cognitive development of their offspring has not been explored.

**Materials and methods:**

Female Sprague–Dawley rats were subjected to chronic maternal sleep interruptions (MSI) during lactation. The offspring were divided into four groups: control, MSI, and MSI with low-dose (5 mg/kg·day) or high-dose (25 mg/kg·day) oleamide (ODA) supplementation. Behavioral performance was assessed using the Morris Water Maze (MWM). Neurogenesis and neuroinflammatory markers in the hippocampus were analyzed through immunohistochemistry, Western blotting, and Q-PCR. Levels of endocannabinoids (eCBs) were measured in maternal milk and offspring brain tissues, along with the expression of eCBs-regulating enzymes in offspring brain tissues. NE-4C cells were used to examine the effects of milk from sleep-disrupted dams on neural function.

**Results:**

Offspring exposed to MSI showed increased escape latency, travel distance, and poor performance in the MWM probe test, indicating impaired spatial learning and memory. MSI also decreased neurogenesis markers and increased neuroinflammatory markers in the hippocampus. High-dose ODA supplementation restored behavioral performance, reduced neuroinflammation, and normalized eCBs levels and enzyme expression in the offspring’s hippocampus. Additionally, MSI altered eCBs composition in maternal milk, particularly lowering ODA and 2-AG levels. *In vitro*, milk from MSI dams inhibited BDNF secretion and reduced anti-inflammatory cytokine expression in NE-4C cells.

**Conclusion:**

MSI during lactation disrupts eCBs signaling and induces neuroinflammation in the offspring, impairing neurodevelopment. Neonatal ODA supplementation may offer a promising intervention to mitigate the cognitive deficits and brain changes induced by MSI during lactation.

## Graphical abstract

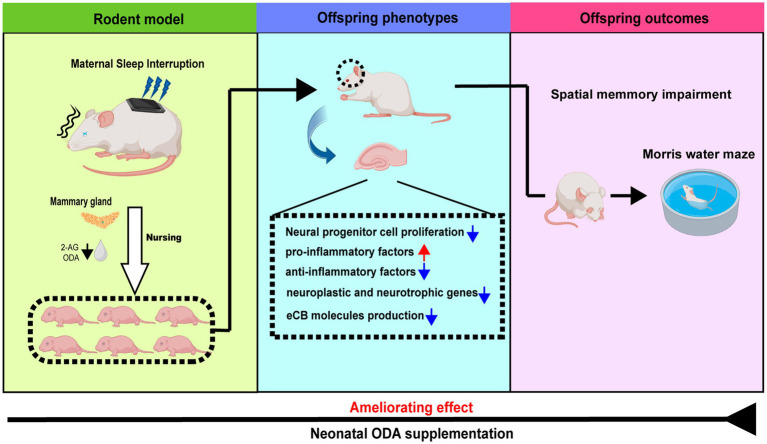

## 1 Introduction

Sleep is a fundamental physiological process that plays a crucial role in overall wellbeing. Prolonged sleep insufficiency can have detrimental effects on mental health, leading to mood disturbances, psychotic symptoms, and cognitive impairments. During the perinatal period, approximately half of gestational women experience disrupted sleep patterns, characterized by hyperarousal, poor sleep quality, sleep fragmentation, and insomnia (1). A meta-analysis conducted by Yang et al. revealed that a significant portion, 67.2%, of postpartum women experienced poor sleep quality (2). This poor sleep quality was strongly influenced by various factors, including frequent nighttime awakenings, perceived stress, marital satisfaction, and the sleep patterns of newborns, all of which contribute to the challenges postpartum women face in achieving restorative sleep (3). A clinical study even indicated that around 50% of individuals who experience clinically significant insomnia symptoms during pregnancy continue to struggle with sleep disturbances even up to 2 years postpartum (4). Although many studies have focused on the adverse effects of sleep loss during pregnancy on infant brain development (5–7), few studies have been conducted on postpartum sleep restriction using animal models. It is essential to explore the molecular and biological mechanisms underlying cognitive deficits in offspring through a rodent postpartum sleep interruption (SI) model, which simulates the sleep fragmentation experienced by breastfeeding women.

The hippocampus, a critical brain region involved in learning and memory processes, is highly susceptible to the effects of stress. A growing body of evidence suggests that maternal sleep deprivation (MSD) can lead to cognitive impairment in offspring, potentially through decreased hippocampal neurogenesis and activation of microglia, the brain’s immune cells (7). Studies have observed an impaired hippocampal long-term potentiation (LTP) and a reduced synaptic transmission in the offspring of MSD rodents (8). Neuroinflammation has been identified as a critical factor in the neurocognitive deficits observed in offspring exposed to MSD. A study by Yao et al. demonstrated that MSD affects the microbial profile in the offspring of MSD-exposed rats, leading to neuroinflammation in the offspring’s brain (9). Furthermore, recent research has shown that MSD disrupts the oxidative and inflammatory balance in the hippocampus through the induction of ferroptosis in rats (10). However, it remains unclear whether postpartum MSD or maternal sleep interruption (MSI) during breastfeeding causes cognitive impairment in offspring.

Breast milk contains numerous neuroactive nutrients that can positively affect brain development and enhance cognitive performance in children. Our previous research has indicated the presence of a group of fatty acid amides (FAMs) in human breast milk (11). These FAMs are characterized by a single amide moiety binding to an alkyl chain. They possess various physiological functions, including analgesic, antianxiety, and anti-inflammatory effects. Among these FAMs, oleamide (ODA) is the most abundant in breast milk, with a concentration of about 1–1.5 mg/L (11). ODA has been well-known for its ability to induce sleep when administered to animal models (12). Our earlier study further demonstrated that supplementing suckling mice with ODA can enhance their cognitive ability and promote neuron proliferation in the hippocampal region after weaning (11).

FAMs (particularly oleoylethanolamide [OEA] and anandamide [AEA]) and 2-arachidonoylglycerol (2-AG) can act as agonists on the endocannabinoid (eCB) system in the brain, mediating various neurobehavioral effects (13). Of these, AEA and 2-AG are the most well-researched eCBs. Despite having similar chemical structures, the synthesis and metabolism of these compounds occur through distinct pathways. N-acyl phosphatidylethanolamine-specific phospholipase D (NAPE-PLD) and diacylglycerol lipase (DAGL) are involved in the synthesis of AEA and 2-AG, respectively, while their degradation is mediated by fatty acid amino hydrolase (FAAH) and monoacylglycerol lipase (MAGL) (14). While ODA is not a classical member of the eCB family, it exhibits cannabimimetic actions similar to AEA and 2-AG, potentially interacting with proteins responsible for eCB biosynthesis, activity, and inactivation (15, 16). The eCB system is integral to neuroplasticity and neurogenesis, influencing learning and memory processes (17). Both direct stimulation of the eCB system by agonists and indirect inhibition of FAAH or MAGL have been shown to impact cognition in various animal models (18–20).

With its ability to activate the CB1 receptor, ODA has demonstrated cognition-enhancing effects in both animal studies and human clinical trials (11, 21, 22). In our current study, we aimed to evaluate the therapeutic potential of ODA in mitigating cognitive impairments observed in the offspring of mothers who experienced sleep disruption (SI) during breastfeeding. We further investigated whether ODA administration could promote neurogenesis, restore balance to inflammatory processes, and consequently improve cognitive-behavioral deficits in these offspring. Additionally, we examined the effects of postpartum SI on the concentration of eCBs in maternal milk and the eCB system in the offspring’s brain. The biological activity of milk from these SI-affected dams, specifically its role in regulating neurogenesis and neuroinflammation, was also assessed using *in vitro* neural stem cell (NSC) models.

## 2 Materials and methods

### 2.1 Animals

Male and female Sprague–Dawley rats were obtained from Shanghai Jihui Laboratory Animal Care Co. To facilitate breeding, two female rats were housed with one male. Mating success was confirmed the next morning by checking for the presence of a vaginal plug. Once pregnancy was verified, each dam was individually placed in a ventilated cage maintained at a stable temperature (23–25°C) with a 12-h light/12-h dark cycle. Animals had unrestricted access to standard grain-based chow and water. On gestational day 16 (GD16), SI vibrators were affixed to the dams. After parturition, pups remained with their biological mothers from postnatal day 0 (PD0) to PD21. To ensure equal nutritional intake across litters, the number of pups per dam was standardized to six. The study protocol was reviewed and approved by the Institutional Review Board and the Animal Care and Use Committee of Shanghai Xinhua Hospital.

**Figure 1:**
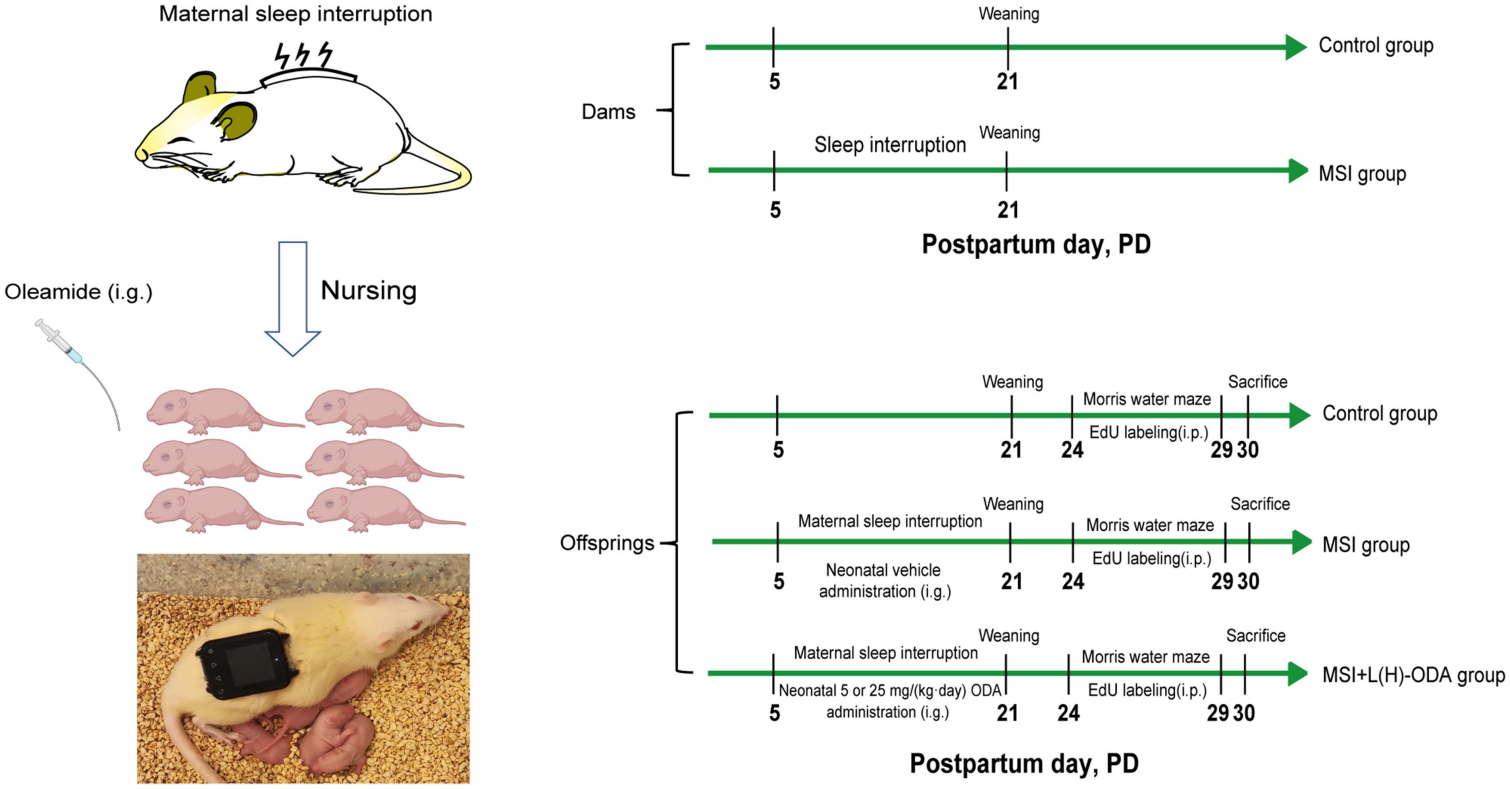
A diagram of the experimental setup. i.p., intraperitoneal injection; i.g., intragastric administration; EdU, 5-ethynyl-2-deoxyuridine; L-ODA, 5 mg/(kg · day) oleamide; H-ODA, 25 mg/(kg · day) oleamide.

Following birth, litters were randomly assigned to one of four groups (six dams per group): (1) Control, (2) MSI, (3) MSI with ODA supplementation at 5 mg/kg/day (MSI + L-ODA), and (4) MSI with ODA supplementation at 25 mg/kg/day (MSI + H-ODA). The MSI procedure was performed on the lactating dams according to the chronic SI protocol which is provided in the supplementary materials. Briefly, the repetitive cycle of vibration was set to 10 s-on and 290 s-off periods in one cycle of 5 min throughout the 24-h period each day from PD5 to PD21. The dams in the control group were put on the same vibrators without vibration. ODA was dissolved in normal saline containing 5% ethanol to prepare a 5 mg/mL solution, administered by oral gavage daily from PD3 to PD21. Pups in the MSI group received an equivalent volume of vehicle solution (saline + 5% ethanol). The ODA dose in this study was determined based on the dosage used in our previous study, which was set at 5 mg/(kg body weight · day) as low dose and 25 mg/(kg body weight · day) as high dose (11). The body weight of pups was recorded on PD3, PD7, PD14, and PD21. On PD21, the dams were separated from the offspring and the milk samples were collected according to a published procedure (23). About 1 ~ 1.5 mL milk per animal can be collected. After leaving enough milk samples for spectrometry analysis, the residual milk samples from either control dams or SI dams were pooled. Afterward, the offspring were weaned onto a standard diet and underwent a water maze test. During behavioral testing, rats received daily intraperitoneal injections of 5-ethynyl-2′-deoxyuridine (EdU) at 50 mg/kg for six consecutive days. Animals were euthanized via cervical dislocation 24 h after the final EdU injection, and hippocampal tissues were quickly dissected. A diagram outlining the overall experimental timeline is provided in Figure 1.

### 2.2 Cell culture and treatment

NE-4C neuroectodermal NSCs (ATCC, USA) were cultured in minimum essential medium (ThermoFisher Scientific, USA), containing 10% fetal bovine serum (HyClone Laboratories, USA), 0.1 mM non-essential amino acids, 2 mM glutamine, and 1% penicillin/streptomycin antibiotic mixture (HyClone Laboratories), in a humidified 5% CO_2_/95% air atmosphere at 37°C. Cells were untreated (non-milk Ctrl) or treated with 1% milk from control dams (Ctrl milk), and 1% milk from SI dams (SI milk). After 24 h treatment, the cells were subjected to ELISA assay and real-time PCR.

### 2.3 Quantification of ODA, AEA and 2-AG in milk and tissue samples

50 mg of tissue or 50 μL of milk was added to 800 μL of methanol. The mixture was vortexed for 30 s and incubated in liquid nitrogen for 1 min. The samples were then thawed and sonicated for 10 min. This process of freezing in liquid nitrogen followed by sonication was repeated three times to ensure thorough sample lysis. To precipitate proteins, the samples were incubated at −20°C for 1 h, followed by centrifugation at 13,000 rpm for 15 min at 4°C. The supernatant was carefully collected and evaporated to dryness. A Waters UPLC BEH C18 column (100 mm × 2.1 mm, 1.7 μm) maintained at 40°C was used for separation by a UPLC system (Acquity, Waters, USA) directly coupled to a TQ-MS (Xevo TQD, Waters). All the standard chemicals were purchased from Sigma-Aldrich (USA). A 1.0 μL sample was injected at a flow rate of 0.35 mL/min. Mobile phase A consisted of water with 0.1% formic acid, whereas mobile phase B consisted of acetonitrile with 0.1% formic acid. The optimized gradient of mobile phase B was as follows: 0–0.5 min, 5% B; 0.5–1.5 min, 5% B; 1.5–3 min, 15–40% B; 3–5 min, 40–70% B; 5–7 min, 70–100% B; 7–8.4 min, 100% B; 8.4–10 min, 100–5% B. Mass spectrometry was conducted using a TQ-MS system equipped with an electrospray ionization ion source in positive (ESI+) ion mode. The system control of the devices and the data acquisition were performed using MassLynx NT 4.1 software (Waters). The operating parameters of the MS analysis were set as: capillary voltage, 2 kV for the positive ion mode; source temperature, 115°C; desolvation gas temperature, 450°C; desolvation gas flow, 900 L/h^−1^; cone voltage, 40 V; reverse cone gas flow, 50 L/h^−1^. The concentrations of ODA, AEA and 2-AG were quantified using calibration curves of respective standards.

### 2.4 Morris water maze test (MWMT)

MWMT was used to assess spatial learning and memory in offspring rats over six consecutive days (PD24 to PD29). A circular water tank (120 cm diameter, 50 cm height) was used with four visual cues placed on its inner walls. The water temperature was maintained at 25°C ± 1°C. Animals were familiarized with the environment for at least 24 h before starting the acquisition phase. The maze was divided into four quadrants: southwest (SW), northwest (NW), northeast (NE), and southeast (SE), with a submerged circular platform (10 cm × 10 cm) placed in the SW quadrant. During the visible platform phase, rats were trained to swim and find the visible platform, with each rat undergoing four trials a day. If a rat did not find the platform within 60 s, it was guided to the platform and allowed to stay for 15 s. The subsequent 4 days involved the hidden platform phase, where the platform was submerged 1 cm below the water surface. The tank was made opaque by adding non-toxic ink to the water, and rats were trained to locate the hidden platform using spatial cues. A video camera was used to record the rats’ movements, and parameters like escape latency, travel distance, and swimming speed were tracked. On the final day (Day 6), a probe test was conducted where the platform was removed, and the rats’ ability to recall the platform location was measured by the time spent in the target quadrant and the number of times they entered the platform’s previous location.

### 2.5 Total RNA isolation and quantitative real-time polymerase chain reaction (qPCR)

Trizol reagent (RNAiso Plus) was used to extract the total RNA of hippocampal tissues. For NE-4C cells, the untreated and treated cells were washed 3 times with PBS, and total RNA was extracted using Trizol reagent. The qPCR was performed as described previously (24). The primer sequences are provided in Supplementary Table 1. The housekeeping gene *β-actin* was used for normalization. qPCR results were analyzed using the 2^−ΔΔCt^ method.

### 2.6 Western blot analysis

Hippocampal tissues were separated on dry ice for immunoblotting and then lysed using radioimmunoprecipitation assay (RIPA) buffer (Beyotime Biotechnology, China) supplemented with a 1% protease inhibitor mixture (Beyotime Biotechnology) and 1% phosphatase inhibitor mixture (Yeasen). A total of 45 μg of protein was separated by SDS-PAGE using a miniature vertical gel electrophoresis (Tanon, Shanghai, China). The gels were then blotted onto a polyvinylidene difluoride (PVDF) membrane (Millipore, USA). After blocking in 5% fat-free milk, the membranes were incubated overnight at 4°C with synaptophysin (SYP, Cell Signaling Technology, USA), postsynaptic density protein 95 (PSD95, Cell Signaling Technology), cyclooxygenase-2 (COX-2, Proteintech, USA), monoacylglycerol lipase (MAGL, Proteintech), fatty acid amide hydrolase (FAAH, Proteintech), NAPE-PLD (Santa Cruz Biotechnology, USA), DAGL (Santa Cruz Biotechnology), nuclear factor-κB (NF-κB, Beyotime Biotechnology), p(Ser 536)-NF-κB (Beyotime Biotechnology), brain-derived neurotrophic factor (BDNF, Abcam, USA) and β-actin (Beyotime Biotechnology) primary antibody. Western blotting was performed and analyzed as described previously (24).

### 2.7 Immunofluorescence staining

Brain tissues were carefully harvested and fixed in 4% paraformaldehyde at 4°C for 24 h. After fixation, the tissues were embedded in paraffin and cut into 5-μm-thick sections using a microtome (Leica, Germany). To prevent non-specific binding, the sections were blocked with 5% goat serum. Following blocking, the sections were incubated overnight at 4°C with primary antibodies: rabbit anti-Doublecortin (DCX) antibody (Servicebio, China) and rabbit anti-ionized calcium-binding adapter molecule 1 (Iba-1) antibody (Servicebio). For the negative control, sections were incubated with normal rabbit IgG instead of the primary antibodies. After incubation, the sections were washed three times (5 min each) with PBS, followed by a 90-min incubation with preadsorbed Alexa Fluor 488-conjugated anti-rabbit secondary antibodies (Servicebio) in the dark. After additional PBS washes, the sections were counterstained with 1 μg/mL 4′,6-diamidino-2-phenylindole (DAPI, Beyotime Biotech) for 5 min to label cell nuclei. EdU staining was conducted using Click-iT Alexa Fluor 594 Imaging kit (Servicebio) according to manufacturer’s instructions. The stained sections were examined using a fluorescence microscope (DS-Ri2, Nikon, Japan). The EdU labeled, Iba-1 labeled, DCX labeled and EdU&DCX colabeled cells within the dentate gyrus (DG) region of hippocampus were quantitated as described previously (11). Slides were analyzed and assessed by blinded investigators.

### 2.8 ELISA assay

After drug administration, the cellular supernatant of NE-4C cells was collected and centrifuged at 1,000×*g* at 4°C for 10 min. The supernatant was collected and the concentration of BDNF was examined by using ELISA kit (Solarbio, China) according to the protocol.

### 2.9 Statistical analysis

For statistical analyses, GraphPad Prism 9.0 was used. The data are presented as mean ± standard error of mean (SEM). Levene’s test and Kolmogorov–Smirnow test were used to analyze the homogeneity of variance and Gaussian distribution of the data. Two-factor repeated-measures ANOVA was performed to compare escape latency, travel distance, swim speed and body weight among groups. The differences in other indices among groups were analyzed using 1-factor ANOVA. Tukey’s test was used to assess the statistical significance between groups following ANOVA tests. A *p* value < 0.05 was considered to be statistically significant.

## 3 Results

### 3.1 ODA attenuates postpartum MSI-induced memory impairment in weaned offspring

**Figure 2:**
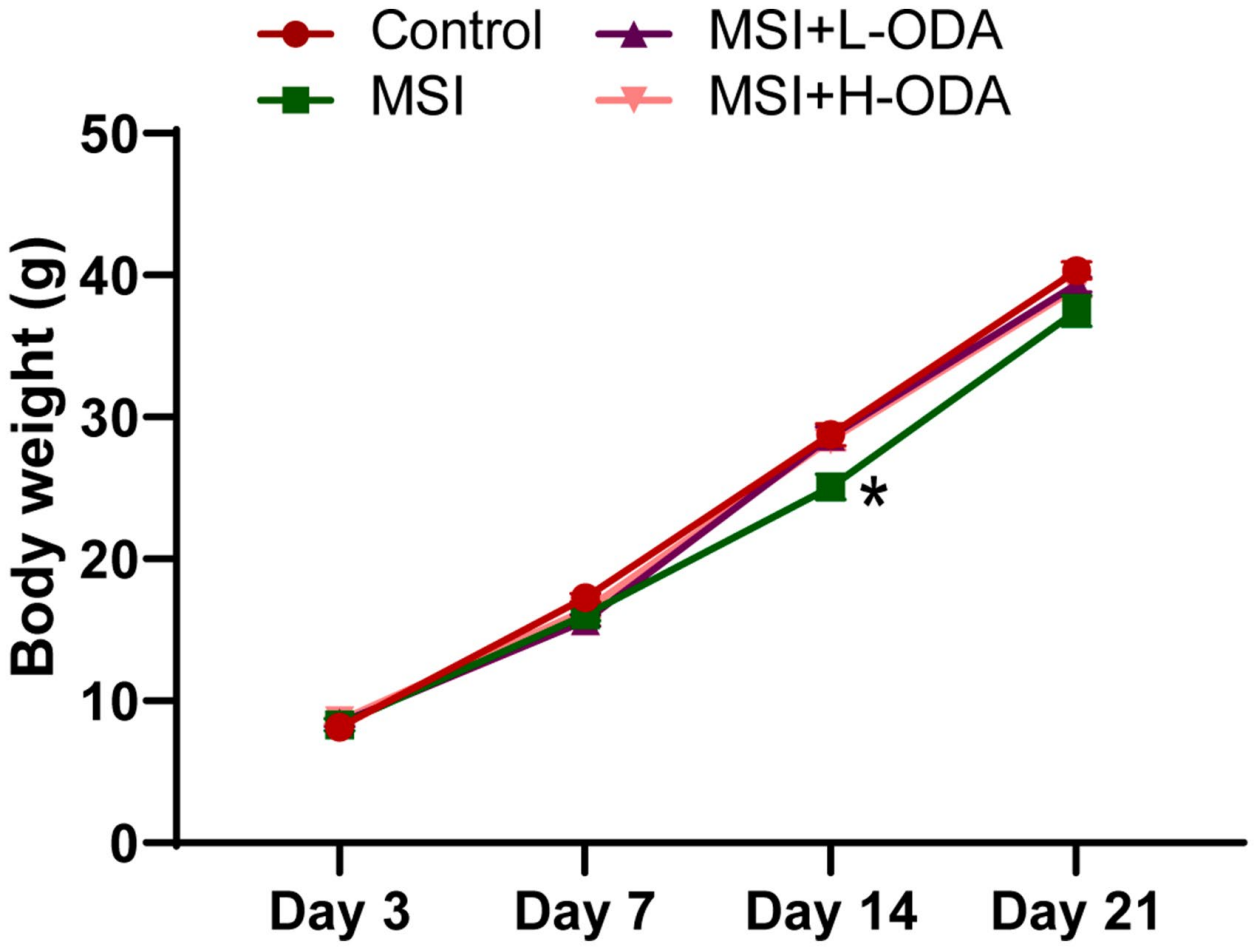
Effects of neonatal ODA supplementation on body weight of MSI–exposed offspring rats. *n* = 7–8 rats per group. Data are presented as the mean ± SEM. *p < 0.05 versus the control group. L-ODA, 5 mg/(kg · day) oleamide; H-ODA, 25 mg/(kg · day) oleamide; MSI, maternal sleep interruption.

To determine whether MSI during lactation affected the growth of suckling pups, we monitored their body weight weekly. As shown in Figure 2, although MSI-exposed dams had offspring with reduced body weight on PD14 (12% decrease; *p* < 0.05), there was no significant difference between the MSI and control groups by PD21. The overall body weight changes during the sucking period showed no significant differences between these two groups. Treatment with both low and high doses of ODA restored the body weight of pups to normal levels by PD14.

**Figure 3:**
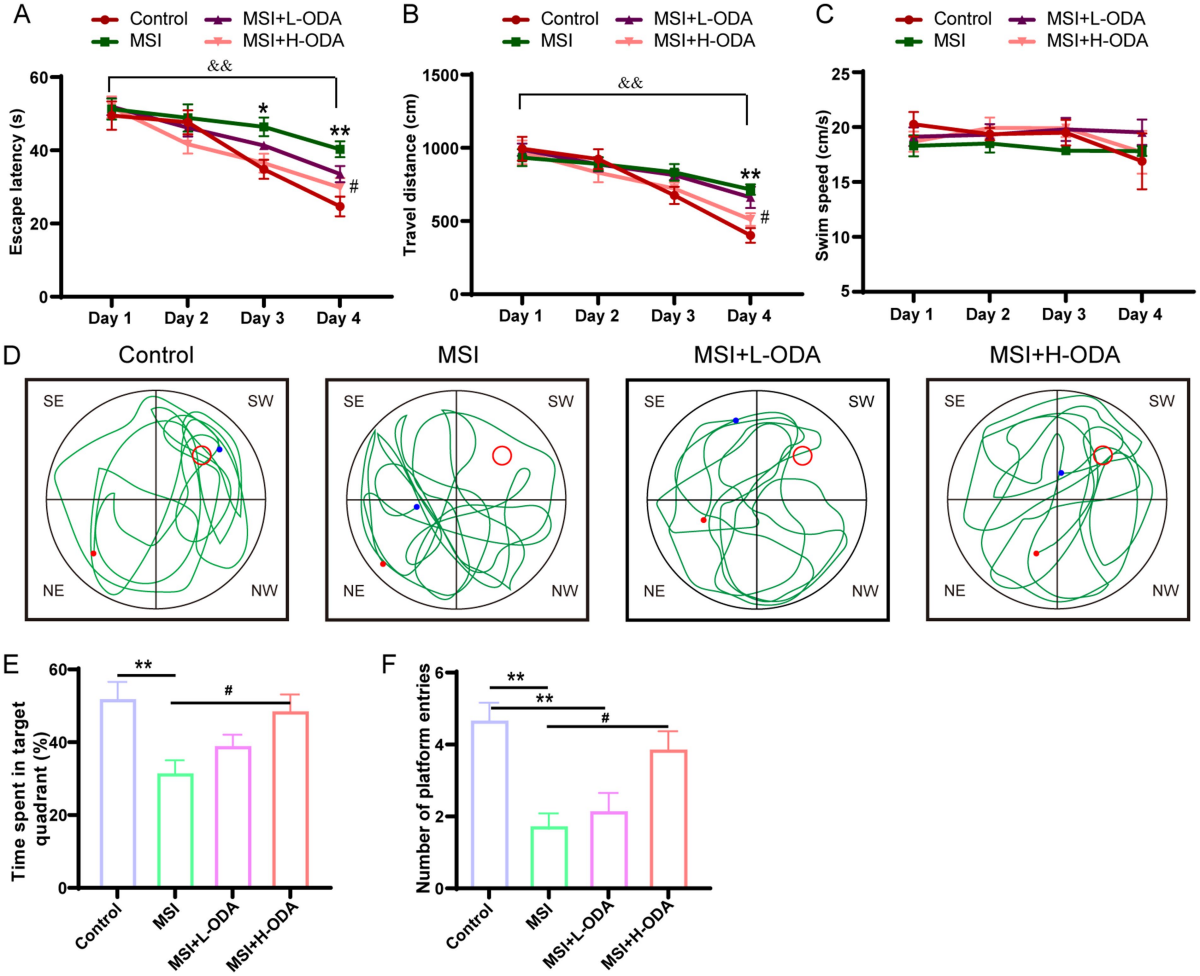
Effects of neonatal ODA supplementation on MWMT performance of MSI–exposed offspring rats. **(A)** Latency to escape, **(B)** distance traveled, and **(C)** swimming speed of rats in the hidden platform phase. **(D)** Example trajectory, **(E)** time spent in target quadrant, and **(F)** number of platform entries of rats from different groups during the probe test. Data are expressed as mean ± SEM; *n* = 6–7 per group. *p < 0.05 and **p < 0.01 for the comparison of treatment effects versus control group; #*p* < 0.05 for the comparison of treatment effects versus MSI group. &&*p* < 0.01 for the comparison of time effects between day1 and day4 within each group. MWMT, Morris water maze test; L-ODA, 5 mg/(kg · day) oleamide; H-ODA, 25 mg/(kg · day) oleamide; MSI, maternal sleep interruption.

The spatial learning and memory performance of young offspring, including those in the control group, MSI group, MSI + L-ODA group, and MSI + H-ODA group, were evaluated using the MWMT. Compared to the control group, the MSI group exhibited a significant increase in escape latency on PD3 and PD4 (33 and 63% increase, respectively; *p* < 0.05) (Figure 3A). Additionally, the MSI-exposed rats showed a significantly higher travel distance than the control group on PD4 (78% increase, *p* < 0.01) (Figure 3B). Treatment with H-ODA restored escape latency and travel distance on PD4 to levels similar to the control group (*p* < 0.05). However, no significant improvements were observed in the L-ODA group compared to the control group. Importantly, there were no significant differences in swimming speed among the groups, indicating comparable motor abilities during the training period (Figure 3C). Following the training phase, a probe test was conducted with the platform removed (Figure 3D). As shown in Figures 3E,F, MSI-exposed rats spent less time in the target quadrant and made fewer platform entries than control rats (40 and 68% decrease, respectively; *p* < 0.01), indicating impaired memory in the MSI group. In contrast, H-ODA treatment significantly increased the time spent in the target quadrant and the number of platform entries compared to the MSI group (54 and 154% increase, respectively; *p* < 0.05). No significant effects on these indices were observed in the L-ODA group. The detailed two-way ANOVA results of the behavioral experiments are provided in Supplementary Table 2. From the results, there was no significant difference in the outcome of MWMT between the MSI and the MSI + L-ODA groups. Thus, we chose high dose (25 mg/kg body weight · day) as a relatively optimal dosage of ODA supplementation for subsequent investigations.

### 3.2 ODA attenuates postpartum MSI-induced inhibition of neurogenesis in weaned offspring

**Figure 4:**
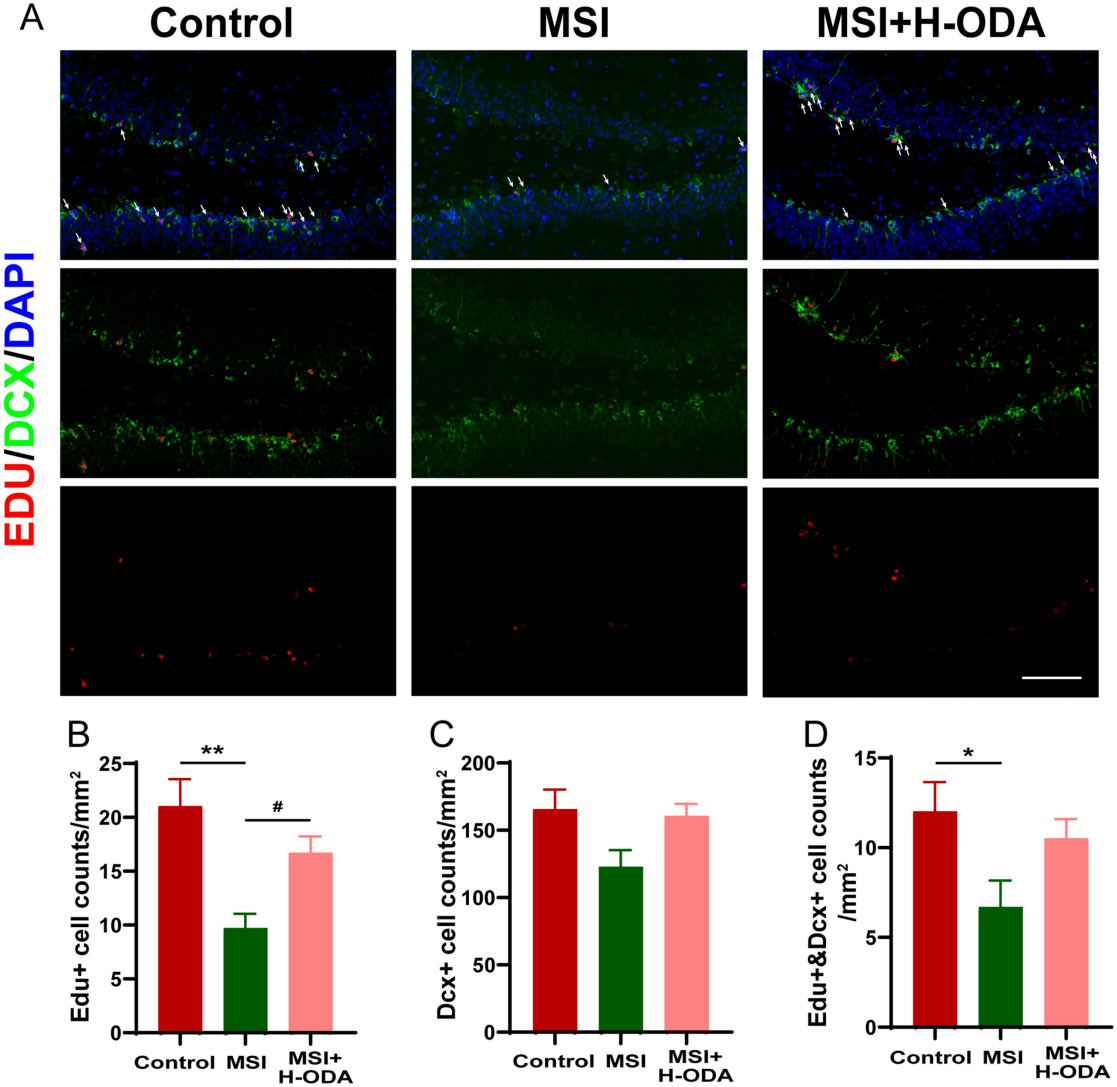
Effects of neonatal ODA supplementation on hippocampal neurogenesis of MSI-exposed offspring rats. **(A)** Representative immunofluorescence images displaying the staining of DCX (green) and EdU (red), with merged images showing EdU-labeled cells indicated by white arrows in the DG region of the hippocampus from different groups. Scale bar: 100 μm. Quantification of the cell density: **(B)** EdU+, **(C)** DCX+, and **(D)** EdU+ &DCX+ cells in the hippocampal DG region for each group. Data are presented as mean ± SEM; *n* = 6 per group. *p < 0.05 and **p < 0.01 versus the control group; #*p* < 0.05 versus the MSI group. DAPI, 4,6-diamidino-2-phenylindole; DG, dentate gyrus; DCX, doublecortin; EdU, 5-ethynyl-2′-deoxyuridine; MSI, maternal sleep interruption; H-ODA, 25 mg/(kg · day) oleamide.

The assessment of neurogenesis in the hippocampus involved the utilization of immunofluorescence labeling with EdU, a marker for proliferative cells, along with DCX, a marker specific to newly formed neurons (Figure 4A). It was found that postpartum MSI significantly reduced neuronal proliferation in the DG region of hippocampus. Specifically, the density of EdU+ cells in the MSI group was 55% lower than in the control group (*p* < 0.01), indicative of a significant decrease in cell proliferation (Figure 4B). In contrast, the density of DCX+ cells did not differ significantly between the MSI and control groups (Figure 4C), suggesting that the differentiation of neural progenitor cells (NPCs) remained relatively unchanged. However, the co-labeling of EdU and DCX revealed a 45% reduction in the number of proliferative NPCs per unit area in the MSI group relative to controls (*p* < 0.05, Figure 4D), pointing toward a decline in the population of proliferating cells that are committed to becoming neurons. Neonatal H-ODA administration restored the EdU+ cell counts and the EdU+ & DCX+ cell counts to levels that were not significantly different from those of the control group.

### 3.3 ODA attenuates postpartum MSI-induced reduction of plasticity-associated proteins and BDNF in weaned offspring

**Figure 5:**
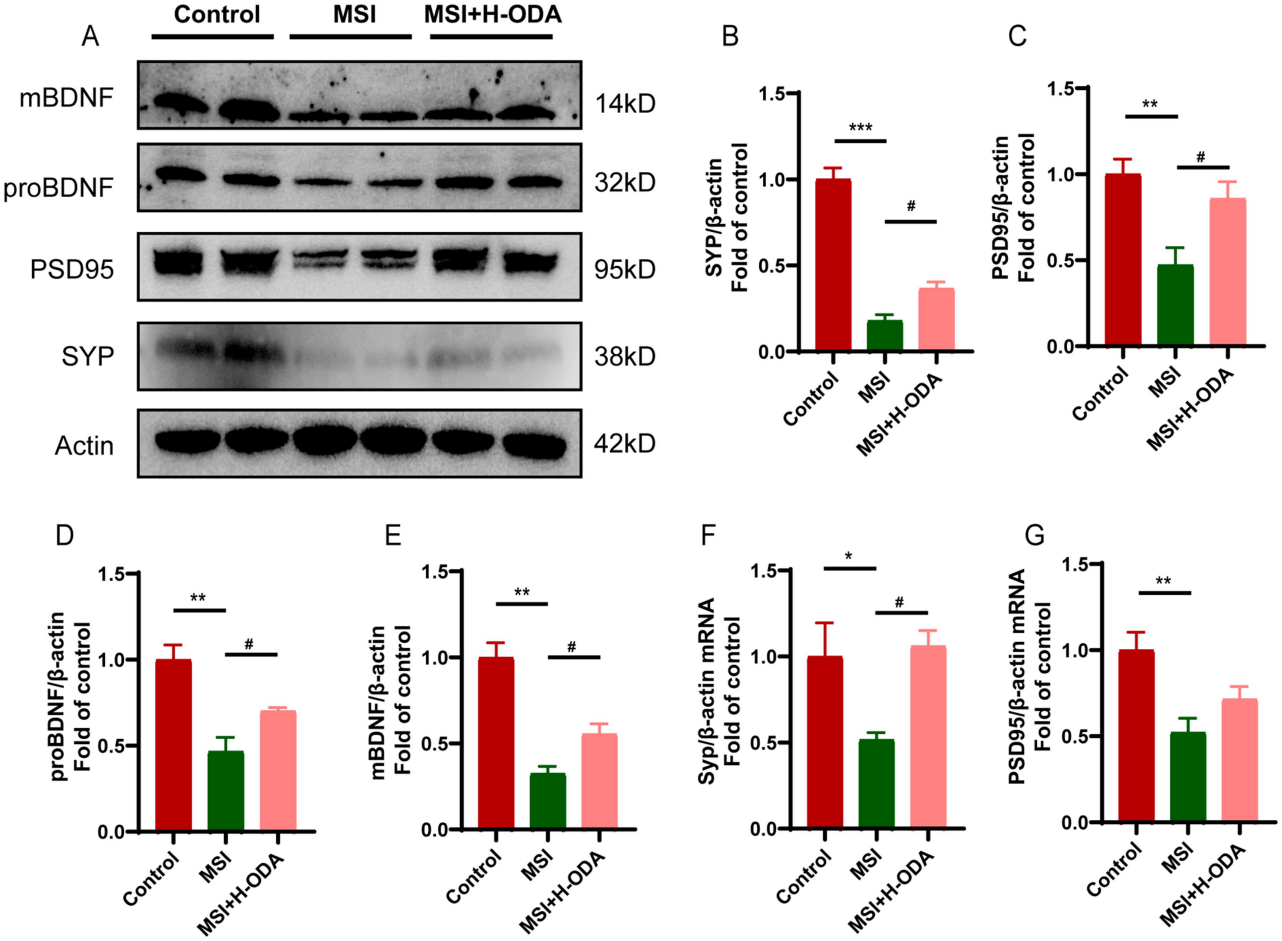
Effects of neonatal ODA supplementation on expressions of synaptic proteins and BDNF in hippocampus of MSI-exposed offspring rats. **(A)** Western blotting of SYP, PSD95, and BDNF expressions in the hippocampus of rats from different groups. Relative band intensity analysis of **(B)** SYP, **(C)** PSD95, **(D)** pro-BDNF, and **(E)** mature BDNF in the samples. **(F,G)** Relative mRNA expressions of *Syp* and *Psd95* in the hippocampus of rats from different groups. Quantification of protein expression was normalized to *β*-actin; *n* = 4–5 rats per group. Quantification of mRNA expressions was normalized to *β-actin* mRNA abundance; *n* = 7 rats per group. Data are presented as mean ± SEM. *p < 0.05, **p < 0.01, and ***p < 0.001 versus the control group; #*p* < 0.05 versus the MSI group. H-ODA, 25 mg/(kg · day) oleamide; MSI, maternal sleep interruption; SYP, synaptophysin; PSD95, postsynaptic density protein 95; BDNF, brain-derived neurotrophic factor.

Western blot analysis revealed significantly lower expression of SYP protein and PSD95 protein in the hippocampus of the MSI group compared to the control group (Figures 5A–C) (79 and 53% decrease, *p* < 0.001 and *p* < 0.01, respectively). Additionally, the mRNA levels of *Syp* and *Psd95* in the MSI group were also significantly lower than those in the control group (Figures 5F,G) (49 and 48% decrease, *p* < 0.05 and *p* < 0.01, respectively). However, neonatal H-ODA administration mitigated the reductions of these synaptic proteins and the reduction of *Syp* mRNA in the hippocampus of MSI-exposed offspring rats. BDNF is a widely recognized neurotrophin found in the brain, promoting neurogenesis and supporting neuroplasticity. To investigate whether the brain impairment of postpartum MSI and the protective effect of ODA were mediated by BDNF, we examined BDNF expression in the hippocampus of three groups. The protein expressions of pro-BDNF and mature BDNF were found to be decreased by postpartum MSI (Figures 5D,E) (54 and 68% decrease, respectively; *p* < 0.01). However, neonatal H-ODA administration mitigated the reductions of these two BDNF isoforms in the hippocampus of MSI-exposed offspring rats (*p* < 0.05).

### 3.4 ODA attenuates postpartum MSI-induced neuroinflammation in weaned offspring

**Figure 6:**
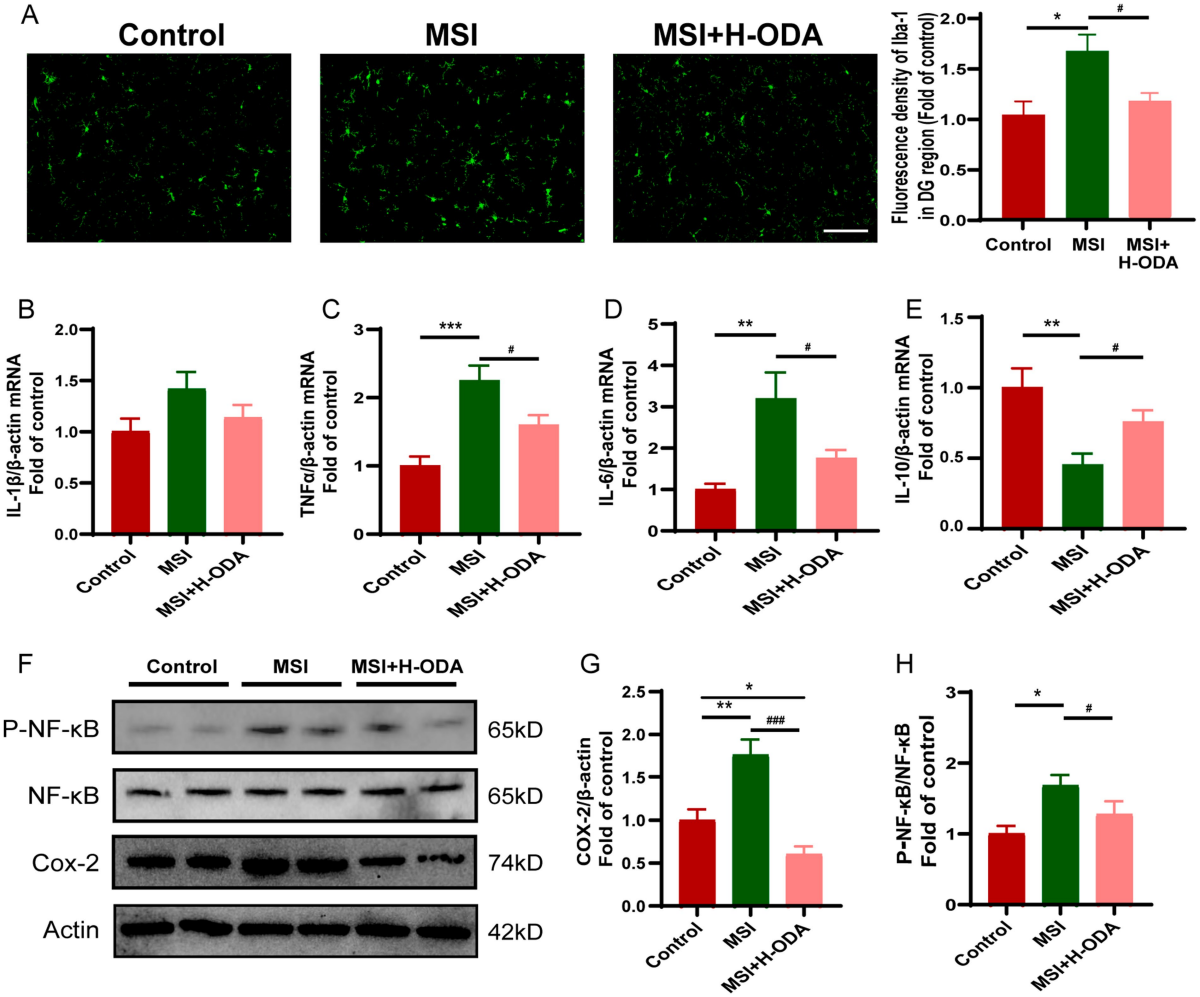
Effects of neonatal ODA supplementation on neuroinflammation in hippocampus of MSI-exposed offspring rats. **(A)** Representative immunofluorescence images illustrating Iba-1 staining in the DG region of the hippocampus from different groups. Scale bar: 100 μm. The fluorescence density of Iba-1 was calculated in the hippocampal DG region of rats from different groups. **(B–E)** Relative mRNA expressions of *Il-1β, Il-6*, *Tnf-α*, and *Il-10* in the hippocampus of rats from different groups. Quantification of mRNA expressions was normalized to *β-actin* mRNA abundance; *n* = 7 rats per group. **(F)** Western blotting of COX-2, p-NF-κB, and NF-κB expressions in the hippocampus of rats from different groups. Relative band intensity analysis of **(G)** COX-2 and **(H)** p-NF-κB in the samples. Quantification of COX-2 expression was normalized to β-actin; Quantitation of the phosphorylation level of NF-κB was normalized to total NF-κB protein. *n* = 4–5 rats per group. Data are presented as mean ± SEM. *p < 0.05, **p < 0.01, and ***p < 0.001 versus the control group; #*p* < 0.05 and ###*p* < 0.001 versus the MSI group. H-ODA, 25 mg/(kg · day) oleamide; MSI, maternal sleep interruption; COX-2, cyclooxygenase-2; Il-6, interleukin 6; Il-1β, interleukin 1 beta; Il-10, interleukin 10; NF-κB, nuclear factor-κB; Tnf-α, tumor necrosis factor alpha.

To investigate whether neuroinflammation contributes to postpartum MSI-related learning and memory impairment, the activity of microglia was assessed using immunofluorescence labeling with Iba-1. The results revealed a significant 63% increase in Iba-1 immunoreactivity in the MSI group compared to controls (*p* < 0.001), indicating heightened microglial activation. This enhanced immunoreactivity was attenuated by neonatal H-ODA supplementation (*p* < 0.05) (Figure 6A). Additionally, real-time PCR was used to analyze changes of proinflammatory and anti-inflammatory cytokines in the hippocampus of offspring rats after MSI. As depicted in Figures 6B–D, the levels of proinflammatory cytokines *Tnf-α* and *Il-6* were significantly increased in the MSI group (128 and 228% increase, *p* < 0.001 and *p* < 0.01, respectively), while H-ODA treatment attenuated their increases (*p* < 0.05). The mRNA level of *Il-1β* in the MSI group was also increased (42% increase); however, not reaching statistical significance when compared with the control group. The mRNA expression of *Il-10*, an important anti-inflammatory cytokine, was significantly decreased in the MSI group (56% decrease, *p* < 0.01), while H-ODA treatment attenuated its decrease (*p* < 0.05) (Figure 6E). Western blot analysis of the offspring hippocampus revealed that the expression of cyclooxygenase (COX)-2, a key inflammatory protein, was significantly higher in the MSI group compared to the control group (77% increase, *p* < 0.01). Notably, H-ODA supplementation reduced COX-2 expression to levels even lower than those observed in the control group (*p* < 0.001) (Figures 6F,G). Because NF-κB is a main transcription factor in the expression of inflammatory cytokines and COX-2 gene (25), we next investigated whether NF-κB pathway participated in the neuroprotection of ODA in MSI caused brain inflammation. The phosphorylation level of NF-κB was 69% higher in the MSI group than that in the control group (*p* < 0.05). Neonatal H-ODA supplementation attenuated the enhanced NF-κB phosphorylation in the MSI + H-ODA group in comparison with the MSI group (*p* < 0.05) (Figures 6F,H).

### 3.5 SI dam’s milk inhibits BDNF production and anti-inflammatory cytokine expression in NE-4C stem cells

**Figure 7:**
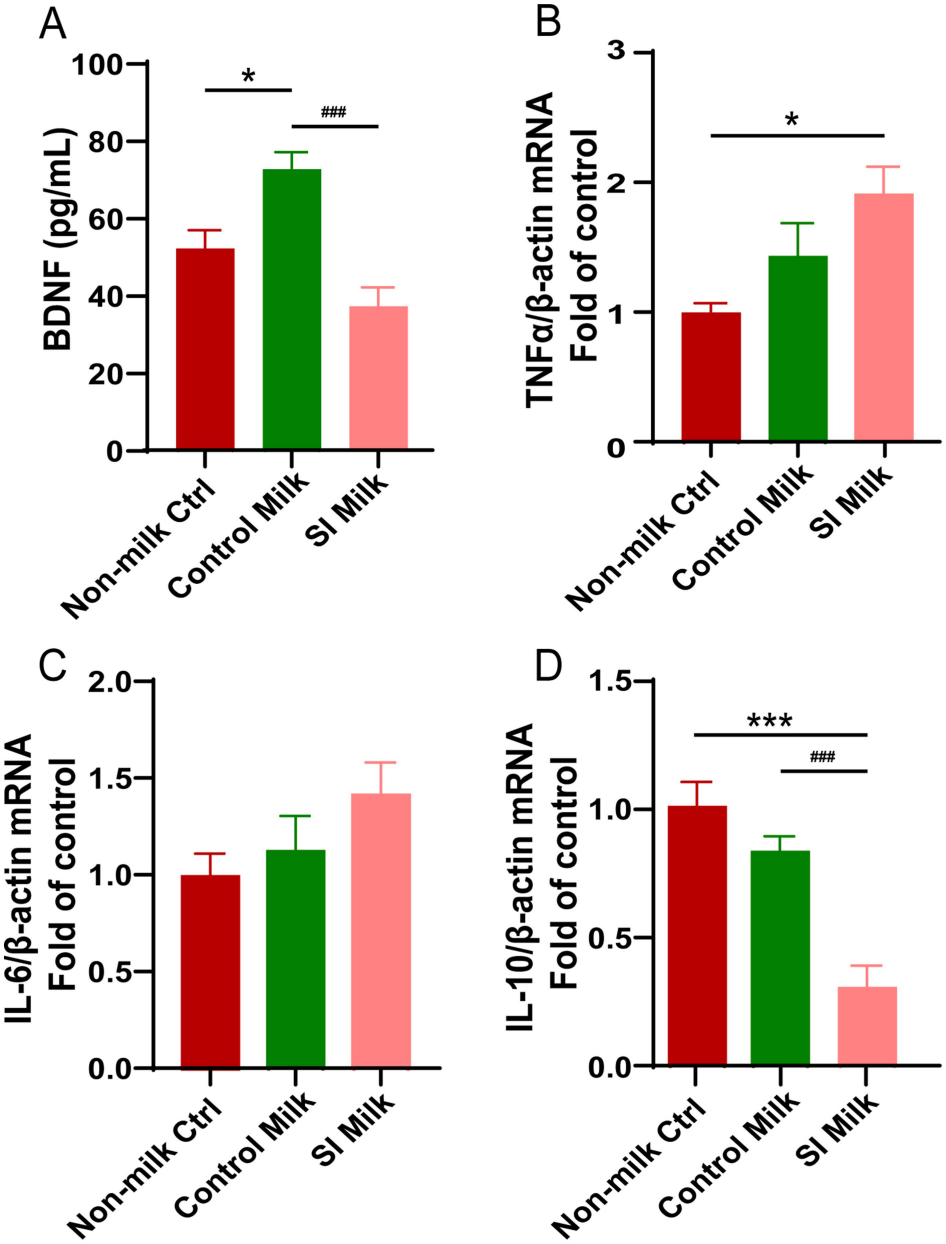
Effects of sleep interrupted dam’s milk on BDNF secretion and (anti- or pro-) inflammatory cytokine expressions in NE-4C stem cells. NE-4C stem cells were untreated (non-milk Ctrl), and treated with either 1% milk from control dams (Control milk) or 1% milk from sleep interrupted dams (SI milk) for 24 h. **(A)** Secretion of BDNF measured by ELISA in supernatant of the untreated and treated cells. **(B–D)** Relative mRNA expressions of **(B)** *Tnf-α*, **(C)** *Il-6*, **(D)** *Il-10* in the untreated and treated cells. Quantification of mRNA expressions was normalized to *β-actin* mRNA abundance; *n* = 6–7 replicates per group. Data are presented as mean ± SEM. *p < 0.05 and ***p < 0.001 versus the untreated cells; ###*p* < 0.001 versus the control milk treated cells. SI, sleep interrupted; Il-10, interleukin 10; Il-6, interleukin 6; BDNF, brain-derived neurotrophic factor; Tnf-α, tumor necrosis factor alpha.

Offspring pups raised by postpartum SI dams exhibited impaired neural development and signs of neuroinflammation compared to those raised by control dams. To explore the role of breast-milk in the transfer of MSI factor to neonatal brain development, we treated the NE-4C stem cells with 1% milk collected from control dams and SI dams. The BDNF concentrations in the supernatants of untreated and milk-treated NE-4C cells were measured by ELISA. As shown in Figure 7A, the control milk increased BDNF secretion by 39% compared to the untreated cells (*p* < 0.05). In contrast to the cells treated with control milk, those treated with SI milk exhibited a 50% reduction in BDNF secretion (*p* < 0.001). Furthermore, the mRNA expression levels of pro-inflammatory and anti-inflammatory cytokines in NE-4C cells were analyzed following treatment with milk from both control dams and SI dams. There was no significant difference in the mRNA levels of the pro-inflammatory cytokines *Il-6* and *Tnf-α* between cells treated with control milk and those treated with SI milk (Figures 7B,C). However, the mRNA level of the anti-inflammatory cytokine *Il-10* was reduced by 64% in cells treated with SI milk compared to those treated with control milk (*p* < 0.001) (Figure 7D).

### 3.6 Postpartum MSI causes reduction of eCBs in dam’s milk and offspring hippocampus

**Figure 8:**
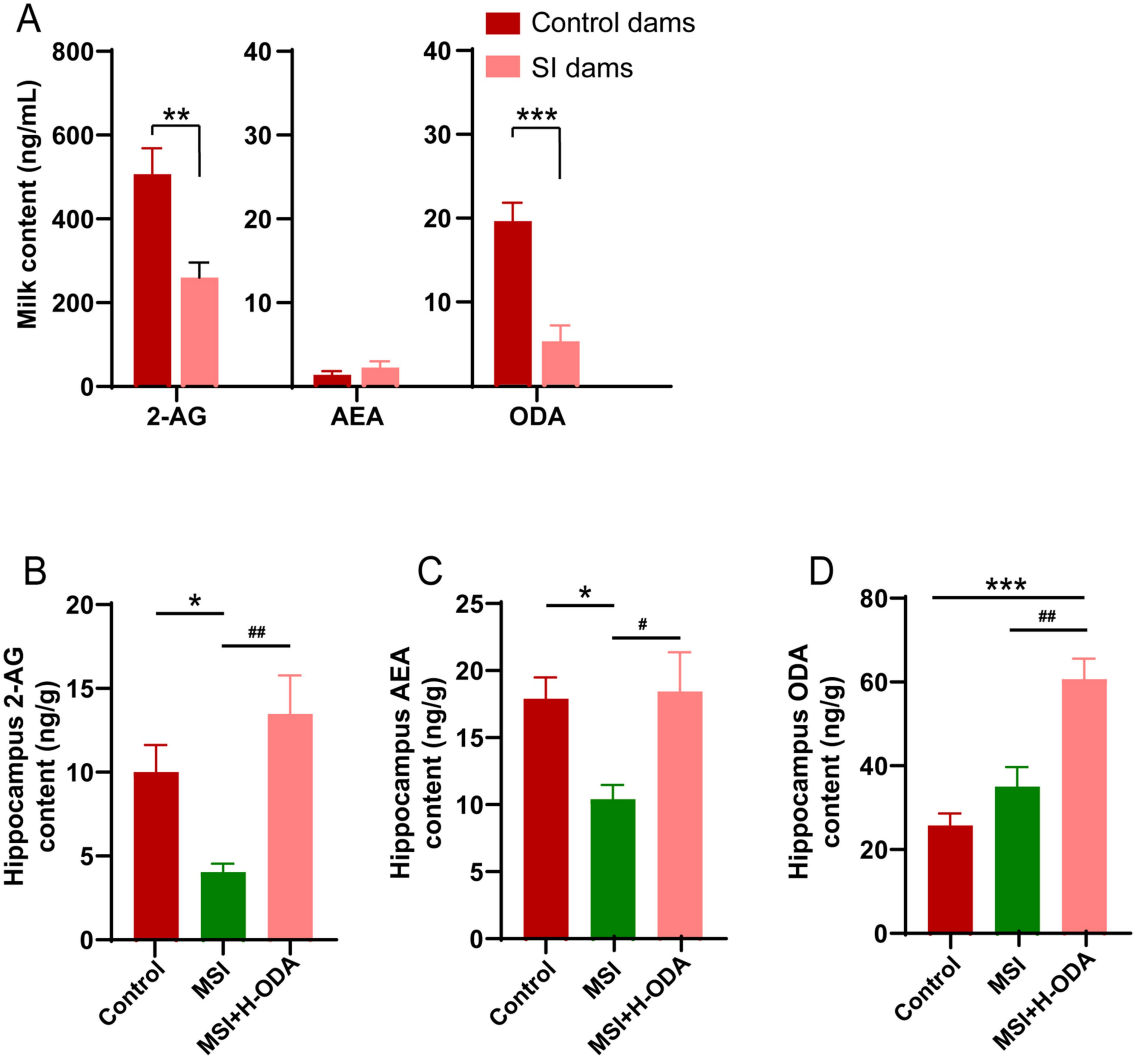
Effects of maternal sleep interruption and neonatal ODA supplementation on endocannabinoid levels in dam’s milk and offspring hippocampus. **(A)** 2-AG, AEA and ODA levels in milk from dams. **(B,C)** 2-AG, AEA and ODA levels in hippocampus of offspring. *n* = 6–7 samples per group. Data are presented as mean ± SEM. *p < 0.05, **p < 0.01 and ***p < 0.001 versus the control group; #*p* < 0.05 and ##*p* < 0.01 versus the MSI group. SI, sleep interrupted; MSI, maternal sleep interruption; AEA, anandamide; 2-AG, 2-arachidonoylglycerol; ODA, oleamide. H-ODA, 25 mg/(kg · day) oleamide.

We measured the levels of three potential eCBs agonists 2-AG, AEA, and ODA in the rat milk on PD21. The results revealed that 2-AG and ODA levels in the milk of SI dams were reduced by 50 and 75% (*p* < 0.01 and *p* < 0.001), respectively, compared to control dams, with no significant difference observed in AEA levels between the two groups (Figure 8A). To explore whether the changes in milk eCBs and neonatal ODA supplementation affected eCBs levels in offspring, we also assessed 2-AG, AEA, and ODA levels in the hippocampus of offspring. As illustrated in Figures 8B–D, the hippocampal levels of 2-AG and AEA in the MSI group were 60 and 42% lower (*p* < 0.05), respectively, than in the control group, while no significant difference was found in hippocampal ODA levels between the two groups. Notably, H-ODA supplementation in MSI-exposed offspring restored hippocampal 2-AG and AEA levels to control levels, while also increasing hippocampal ODA levels by 139% compared to the control group (*p* < 0.001).

### 3.7 ODA attenuates postpartum MSI-induced reduction in eCB synthesis enzymes in offspring hippocampus

**Figure 9:**
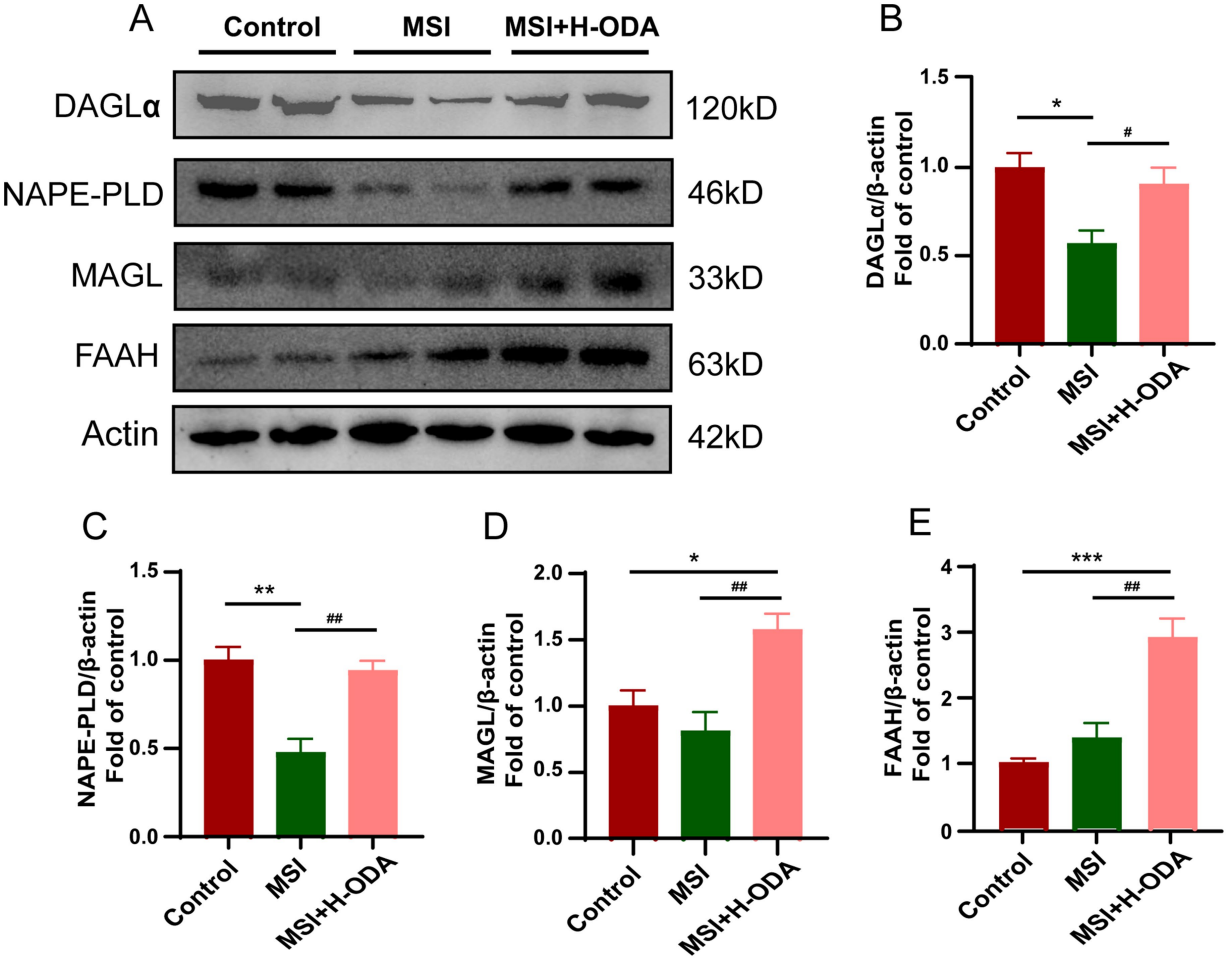
Effects of maternal sleep interruption and neonatal ODA supplementation on expressions of endocannabinoid synthesizing and degrading enzymes in hippocampus of MSI-exposed offspring rats. **(A)** Western blotting of DAGL, NAPE-PLD, MAGL, and FAAH expressions in the hippocampus of rats from different groups. Relative band intensity analysis of **(B)** DAGL, **(C)** NAPE-PLD, **(D)** MAGL, and **(E)** FAAH in the samples. Quantification of protein expression was normalized to β-actin. *n* = 4–5 rats per group. Data are presented as mean ± SEM. *p < 0.05, **p < 0.01, and ***p < 0.001 versus the control group; #*p* < 0.05 and ##*p* < 0.01 versus the MSI group. H-ODA, 25 mg/(kg · day) oleamide; MSI, maternal sleep interruption; DAGL, diacylglycerol lipase; MAGL, monoacylglycerol lipase; FAAH, fatty acid amide hydrolase; NAPE-PLD, N-acyl phosphatidylethanolamine-specific phospholipase D.

The impact of MSI on the expression of key enzymes involved in the synthesis and degradation of eCBs in the hippocampus of weaned offspring was assessed. Western blot analysis demonstrated that MSI significantly decreased the expression of NAPE-PLD and DAGL, which are crucial for the biosynthesis of AEA and 2-AG, respectively (Figure 9A). Specifically, there was a 54% decrease in NAPE-PLD and a 44% reduction in DAGL expression in the MSI group compared to the control group (*p* < 0.05). Treatment with H-ODA effectively counteracted these changes (*p* < 0.05). The NAPE-PLD and DAGL levels in the hippocampus of the MSI + H-ODA group were restored to levels comparable to the control group (Figures 9B,C). Importantly, the expression levels of FAAH and MAGL, responsible for the degradation of AEA and 2-AG, were not significantly altered by MSI. Furthermore, a significant increase in the expression of FAAH and MAGL was observed in the MSI + H-ODA group compared to both the control and MSI groups (Figures 9A,D,E) (*p* < 0.05).

## 4 Discussion

The postpartum period is a critical phase for both the mother and the newborn, as it entails numerous physical, emotional, and physiological changes. Among the various factors that influence this phase, maternal nutrition, sleep quality and breastfeeding practices play pivotal roles. Sleep disturbances are common during the postpartum period, primarily due to the demands of caring for newborn, hormonal fluctuations, and physical discomfort (26). Disrupted sleep patterns, characterized by frequent awakenings and shortened sleep duration, can have profound effects on maternal wellbeing. Sleep disruptions experienced by new mothers can impact their ability to breastfeed effectively, while breastfeeding practices can contribute to further sleep disturbances (27). Both factors, in turn, can influence the infant’s development and wellbeing. Addressing the interrelationship between these two elements are crucial for comprehensive postnatal care. A previous study had reported that lactating women have a marked alteration in their sleep architecture, specifically an enhancement of slow wave sleep, when compared with control and bottle-feeding women. This change of sleep architecture has been suggested as a result of elevated circulating prolactin in breastfeeding women (28). Recent study showed that the maternal sleep duration was shortest (7.02 ± 1.05 h/day) and the incidence of insufficient maternal sleep was highest (34.29%) at 5–7 months postpartum. They demonstrated that the less sleep duration of breastfeeding women may be related to the decreased serum ghrelin concentration (29). Although breastfeeding practices can affect maternal sleep, so far, little is known about the effects of insufficient maternal sleep on lactation process. A study found that shortened postpartum sleep duration may be related to less milk secretion (30). However, no direct evidence has been found to confirm insufficient sleep during breastfeeding can impact milk nutritional constituents.

This study introduces an innovative method for selectively disrupting the sleep of individual animals within a group, offering distinct advantages over traditional sleep fragmentation techniques. In experiments exploring the relationship between sleep fragmentation and normal physiological and social activities, maintaining animals’ natural living conditions is crucial to reduce confounding factors. Existing methods, such as disk-over-water, forced locomotion, gentle handling, and pharmacological sleep deprivation, have limitations (31–36). Disk-over-water and forced locomotion methods remove animals from their normal environment, applying excessive stress that may cause them to reduce activity, making long-term experiments difficult. Gentle stimulation requires manual intervention and is difficult to standardize, while pharmacological approaches introduce side effects that may confound results. Our device addresses these issues by allowing precise sleep disruption without interfering with animals’ ability to engage in normal physiological and social behaviors. Unlike traditional methods, it does not disturb the entire environment or require transferring animals between living and experimental setups. Additionally, the device provides a standardized, programmable method to control the number and duration of sleep fragments, making it suitable for chronic sleep fragmentation studies. According to our results, the chronic SI regimen using our apparatus moderately increased wakeful time in treated rats during the first week of SI, but this difference dissipated after 2 weeks. However, the number of wake bouts and the average sleep bout duration remained significantly higher and lower, respectively, compared to baseline throughout the two-week SI period. These findings demonstrate that the device effectively induces a gentle and tolerable sleep disturbance, particularly in the form of fragmentation, over an extended period, aligning well with the demands of rodent lactation in our subsequent experiments. Although the total sleep time began to recover slightly after 2 weeks of SI, the sleep fragmentation persisted throughout the entire SI process.

Based on the MWMT results, the MSI-exposed offspring rats displayed increased escape latencies and travel distance in comparison to the control group on the later training period. The prolonged parameters implied that the postpartum MSI dampened the acquisition of information from surrounding during learning process in offspring rats, while neonatal H-ODA administration restored their injured learning ability. The attenuated ability of MSI-exposed offspring rats in the probe trial, exhibiting reduced time in the target quadrant and reduced number of platform entries, further confirmed the results obtained from training period. Spatial and contextual memory formation is closely linked to the dorsal hippocampus, particularly the subgranular zone (SGZ) of the DG, which is one of the few brain regions capable of continuous neurogenesis throughout life (37). Cognitive processes, especially those related to memory, are tightly associated with hippocampal neurogenesis, as newly formed NPCs in the SGZ migrate to the outer layers of the DG, where they eventually contribute to cognitive functions (38, 39). In this study, we investigated the relationship between impaired cognitive function due to MSI and hippocampal neurogenesis using EdU and DCX labeling. We observed reduced densities of EdU-labeled cells and a decrease in the number of EdU & DCX double-labeled cells in the hippocampal DG region of MSI-exposed offspring, suggesting that MSI disrupts the proliferation of NPCs. Furthermore, we examined the expression of synaptic plasticity-related markers. PSD95 is crucial for synapse development and functions in postsynaptic membranes and dendritic spines (40), while SYP is a key marker of synapse formation (41). Our findings revealed a reduction in the expression of both of these synaptic plasticity markers in the hippocampus of MSI-exposed rats. The observed reductions in neurogenesis and synaptic protein expressions in the hippocampus correlate with the poor performance in behavioral tests. However, high dose ODA administration was able to reverse these deficits.

While previous studies have primarily focused on the impact of MSD during pregnancy on offspring brain development through mechanisms like maternal systemic inflammation (42), our study examined the effects of MSI during suckling on neuroinflammation in the offspring brain. We demonstrated that MSI during suckling significantly induced neuroinflammatory changes in the hippocampus of weaned offspring, with effects similar to the widely studied MSD model during pregnancy (7, 42). Specifically, MSI during suckling seems to influence offspring neurodevelopment by interfering with maternal care and altering the composition of breast milk, including reductions in ODA and 2-AG levels, both of which are crucial regulators of the endocannabinoid system and neuroinflammatory balance. Our findings revealed that MSI during suckling led to significant activation of microglia in the hippocampus, as evidenced by an increase in Iba-1+ cells. This microglial activation was associated with an upregulation of pro-inflammatory M1 cytokines (TNF-α and IL-6) and a downregulation of the anti-inflammatory M2 cytokine IL-10, promoting a pro-inflammatory environment in the developing brain. These changes are consistent with previous literature linking neuroinflammation to cognitive deficits and impaired neurogenesis (43–46). However, we did not observe an increase in the classical pro-inflammatory cytokine IL-1β in the brains of MSI-exposed offspring. Additionally, MSI also induced elevated phosphorylation of NF-κB, a well-known mediator of inflammatory signaling. This increase in NF-κB phosphorylation further supports the idea that MSI promotes neuroinflammatory pathways, as NF-κB activation is closely associated with microglial activation and the upregulation of pro-inflammatory cytokines (47, 48). Notably, high-dose ODA supplementation effectively counteracted these neuroinflammatory changes. It not only restored abnormal microglial activation and cytokine expression patterns but also normalized the elevated phosphorylation levels of NF-κB, highlighting ODA’s potential to rebalance the neuroimmune response and mitigate microglial activation. These findings align with previous studies suggesting the anti-inflammatory and neuroprotective roles of ODA and other fatty acid amides (49, 50).

In contrast to the *in vivo* findings, our *in vitro* experiments using NE-4C neural stem cells treated with milk from SI-exposed dams revealed a different pattern of cytokine expression. While SI milk significantly reduced IL-10 expression, it failed to induce the expression of pro-inflammatory M1 cytokines TNF-α and IL-6, which were elevated in the *in vivo* hippocampal analysis. This discrepancy could be explained by several factors. First, the *in vitro* environment lacks the complex interactions present in the developing brain, where direct cell–cell communication between neurons, astrocytes, and microglia amplifies inflammatory signals. Additionally, this discrepancy may be attributed to the fact that *in vitro*, NE-4C cells are exposed directly to the components of the milk—such as lipids, extracellular vesicles, and immune regulatory factors—allowing for a direct action on the cultured cells. In contrast, *in vivo*, these milk components are likely altered by digestion before they can influence the developing brain, which may change their effects on neural development. We also observed that SI milk significantly reduced BDNF secretion from NE-4C cells compared to control milk, which is consistent with the *in vivo* findings of reduced BDNF levels in the hippocampus of MSI-exposed offspring. Furthermore, control milk itself enhanced BDNF secretion in NE-4C cells compared to the non-milk control. This suggests that breast milk contains certain neurotrophic factors that can directly promote neural development. MSI in rat dam altered the neurodevelopmental benefits of maternal milk, which may contribute to the impaired cognitive outcomes observed in MSI-exposed offspring. These findings emphasize the critical role of maternal milk in supporting neutral cell development and suggest that sleep fragmentation during lactation may disrupt this process through alterations in milk composition.

In this study, we measured the levels of ODA, AEA, and 2-AG in the milk of dams exposed to both control and SI conditions, as well as in the hippocampus tissues of offspring subjected to control, MSI, and MSI supplemented with high-dose ODA. Our results demonstrated that SI during lactation significantly reduced the levels of specific eCB agonists, namely 2-AG and ODA, in maternal milk. Interestingly, the levels of AEA remained unchanged, suggesting a selective impact of SI on the eCB components of milk. Both ODA and AEA have been identified as sleep inducers (51, 52). ODA was first detected in the cerebrospinal fluid of sleep-deprived cats and later reported to suppress motor activity and induce sedated behavior and eye closing, suggestive of sleep, in rats (53, 54). The comparison between our current data and Basile’s data indicates that the milk ODA level is approximately 100 times higher than its plasma level, suggesting that ODA in milk is synthesized by the mammary gland rather than diffusing from the blood (11, 55). This implies that the fluctuation of ODA concentration in breast milk is likely under tight neuroendocrine regulation by the lactating mammary gland. In the present study, SI during lactation led to the reduction of ODA and 2-AG levels in maternal milk. This could be explained by the possible impact of SI during lactation on dam’s neuroendocrine pathway, resulting in the changes of synthesis or degradation of eCB components in the mammary gland. The current study suggests that sleep fragmentation could interfere with the physiological mechanisms that control milk composition, potentially due to changes in maternal metabolism and neuroendocrine functions, which are known to be influenced by the mother’s sleep quality and anxiety levels (56–61).

Previous research has demonstrated that maternal diet and lifestyle, including factors like circadian rhythms and sleep, can substantially influence milk eCB profiles. For instance, a high-fat maternal diet has been shown to reduce AEA and 2-AG levels in milk, leading to altered cannabinoid signaling and neurodevelopmental outcomes, including increased predisposition to obesity and altered food preferences in offspring (62). Similarly, fluctuations in 2-AG levels in maternal milk, influenced by circadian rhythms, highlight how environmental stressors can modulate the eCB content in milk (63). The eCB system in milk is also essential for offspring neurobehavioral development. Studies by Fride et al. have demonstrated that 2-AG in milk facilitates suckling behavior in neonates, with CB1 receptor activation being crucial for initiating feeding responses (64). Reduced levels of ODA and 2-AG in milk due to MSI could therefore impair these critical neurodevelopmental processes, potentially affecting cognitive and emotional behavior. Furthermore, research by Manduca et al. emphasized that maternal eCB signaling regulates social and emotional development, with disturbances in this pathway affecting early mother-infant bonding and social behaviors (65). Additionally, eCBs have been shown to play a critical role in neurogenesis and synaptic plasticity in the hippocampus, which are vital for cognitive function (66). Consistent with these findings, our study demonstrates that MSI significantly reduced hippocampal 2-AG and AEA levels in offspring, contributing to cognitive deficits. Interestingly, supplementation with high dose ODA restored these levels, suggesting that dietary interventions may help mitigate the adverse effects of MSI and restore eCB balance, as previously suggested by Reyes Prieto et al. (67). This research underscores the importance of maternal eCB signaling in regulating offspring neurodevelopment and suggests that targeted neonatal interventions could optimize eCB communication during lactation.

Dysregulation of eCB signaling has been implicated in the neurobehavioral disturbances. It is well-established that the administration of exogenous cannabinoids can negatively affect short-term memory and cognition in both humans and animals. This impairment is likely due, at least in part, to the suppression of long-term potentiation (LTP) caused by prolonged and widespread activation of CB1 receptors by external agonists (68, 69). On the other hand, the controlled and localized release of endogenous cannabinoid molecules can potentially enhance synaptic plasticity in a more targeted manner (70, 71). The roles of eCBs are multifaceted, as they regulate synaptic activity by inhibiting the release of GABA and glutamate, which in turn has opposing effects on postsynaptic activity (72). Zimmermann’s study used spatially specific FAAH transgenic mice to achieve the diminish of fatty acid amides in hippocampal CA1-CA3 glutamatergic neurons, and that disturbance impaired learning and emotional responses (19).

This study investigated the critical role of the eCB system in cognitive and behavioral development, highlighting the impact of MSI during lactation on the eCB system in the hippocampus of offspring. Our findings revealed that MSI significantly reduced the expression of essential enzymes, NAPE-PLD and DAGL, in the offspring hippocampus, which are pivotal for the synthesis of AEA and 2-AG, respectively (14). Interestingly, the expression of degradation enzymes, FAAH and MAGL, remained unaffected, suggesting that MSI affects eCB levels primarily by impairing synthesis rather than enhancing degradation. This observation indicates that MSI-induced disruptions primarily alter the endogenous eCB metabolism within the offspring’s brain rather than limiting the direct transfer of eCBs through maternal milk. Administration of a high dose of ODA effectively restored the expression of NAPE-PLD and DAGL, thus normalizing eCB levels in the hippocampus. Additionally, high dose ODA administration led to increased expression of FAAH and MAGL, potentially as part of a feedback mechanism to balance the elevated ODA intake, underscoring the complex self-regulating properties within the eCB system.

In this study, the decreased expressions of NAPE-PLD and DAGL in the offspring hippocampus under MSI conditions underscores that maternal sleep quality can affect the eCB synthesis pathway in the developing brain. Critically, studies on the gut-brain axis have shown that dietary and gastrointestinal sources of eCBs, such as those obtained through breast milk, can exert substantial indirect effects on the brain’s eCB system (73, 74). The gut-brain axis facilitates bidirectional communication, whereby dietary eCBs and gut-derived metabolites modulate brain functions and behaviors through intricate neural, hormonal, and immune interactions (75). In our case, while eCBs in milk are metabolized to a degree within the infant’s gastrointestinal system, they still possess the capacity to influence the brain’s eCB system indirectly. This is achieved through the gut-brain axis, where eCBs and related molecules interact with cannabinoid receptors and other cellular pathways along the gut-brain signaling network (75). Such interactions have been implicated in regulating the brain’s eCB system, thus affecting cognitive, emotional, and behavioral responses.

## 5 Conclusion

In conclusion, our study demonstrates that MSI during lactation significantly impairs the cognitive development and neurogenesis of offspring. Specifically, MSI disrupts eCB signaling and induces neuroinflammation in the hippocampus, leading to deficits in spatial learning and memory. The supplementation of ODA during suckling effectively mitigates these adverse effects, restoring hippocampal neurogenesis, synaptic plasticity, and eCB homeostasis. Moreover, ODA supplementation normalizes inflammatory responses and enhances cognitive function in offspring, highlighting its potential as a therapeutic intervention for cognitive impairments induced by maternal sleep disturbances. These findings underscore the importance of maternal sleep quality during lactation and suggest that ODA supplementation may offer a promising strategy to counteract the neurodevelopmental consequences of mother sleep disruption in the early postnatal period.

## Patents

The device used in this manuscript was authorized by Chinese patent office (Chinese patent number 202323373065.4).

## Data Availability

The raw data supporting the conclusions of this article will be made available by the authors without undue reservation.

## Glossary

SI: sleep interruption (or interrupted)
MSI: maternal sleep interruption (or interrupted)
SD: sleep deprivation
MSD: maternal sleep deprivation
LTP: long-term potentiation
FAMs: fatty acid amides
ODA: oleamide
OEA: oleoylethanolamide
AEA: anandamide
2-AG: 2-arachidonoylglycerol
FAAH: fatty acid amide hydrolase
MAGL: monoacylglycerol lipase
eCB: endocannabinoid
CB: cannabinoid
NAPE-PLD: N-acyl phosphatidylethanolamine-specific phospholipase D
DAGL: diacylglycerol lipase
NSCs: neural stem cells
EDU: 5-ethynyl-2′-deoxyuridine
DAPI: 4′,6-diamidino-2-phenylindole
Il-1β: interleukin 1 beta
Il-6: interleukin 6
Il-10: interleukin 10
Tnf-α: tumor necrosis factor alpha
BDNF: brain-derived neurotrophic factor
MWMT: morris water maze test
qPCR: quantitative real-time polymerase chain reaction
DCX: doublecortin
DG: dentate gyrus
SGZ: subgranular zone
NPCs: neural progenitor cells
SYP: synaptophysin
PSD95: postsynaptic density protein 95
COX-2: cyclooxygenase-2
NF-κB: nuclear factor-kappa B
Iba-1: ionized calcium-binding adapter molecule 1
PD: postpartum day
GD: gestational day.
