# Supplementary material for: Neonatal supplementation of oleamide during suckling ameliorates maternal postpartum sleep interruption-induced neural impairment and endocannabinoid dysfunction in early adolescent offspring rats
Source: Front Nutr. 2025 May 12;12:1566182. doi: 10.3389/fnut.2025.1566182 (PMC12104719; doi:10.3389/fnut.2025.1566182)
Supplement: Supplementary file 1 [file Table_1.DOCX]

**Online Supplementary data**

**A new automated and remotely controlled apparatus for rodent sleep interruption**

**Supplementary Methods**

**Animals.** The experiments were carried out on male Sprague-Dawley rats (Shanghai Jihui Laboratory Animal Care Co). The animals were maintained under standard laboratory conditions (12-h light/dark cycle, room temperature at 21± 1 °C) with free access to water and standard grain-based diet in their home cages, and adapted to the laboratory conditions for at least one month.

**Sleep Monitoring.** Under pentobarbital anesthesia (50 mg/kg, i.p.), rats (3 months old) were implanted with electroencephalogram (EEG) and electromyogram (EMG) electrodes. The implant consisted of 2 stainless steel screws (1 mm diameter) inserted through the skull into the cortex (1.0 mm anterior to bregma, 1.0 mm right of the central suture and 1.0 mm posterior to lambda on the extended line of the central suture) and served as EEG electrodes. Two insulated stainless steel, Teflon-coated wires bilaterally placed into both trapezius muscles served as EMG electrodes. All electrodes were attached to a microconnector and fixed to the skull with dental cement. After a 7-day recovery period, animals were subjected to further sleep interruption (SI) experiment.

Cortical EEG and EMG signals were amplified and filtered (EEG at 0.5–30 Hz and EMG at 20–200 Hz), digitized at a sampling rate of 128 Hz, and recorded using MedLab (U/4C501H, Nanjing Yimei, China). When completed, the states of vigilance were automatically scored off-line under 10-s epochs into three stages, i.e., wakefulness, rapid eye movement (REM) and non-rapid eye movement (NREM) sleep, using AccuSleep software. Waking was scored when fast, desynchronized EEG was observed in combination with high EMG-amplitude; NREM sleep was scored when higher amplitude EEG waves were observed and delta (0.5–4 Hz) activity was predominant; and REM sleep was scored when EEG theta (4–7 Hz) activity was predominant while EMG activity was virtually absent (Fig. 3A).

**Sleep interruption.** The SI device was authorized by Chinese patent office (Chinese patent number 202323373065.4), and it is specifically designed for observing breastfeeding and social behaviors in rodents experiencing sleep fragmentation (Fig. 1). This apparatus comprises a programmable time controller (SP-0816MT, Stateframe, China), a wireless transmitter (F801, Tuchuanlu, China), and vibrators (L805, Tuchuanlu, China), as illustrated in Fig. 2A-C. The vibrator was fastened on the back of the rats using metal cable ties and adjusted until a comfortable fit was achieved. The experimental rats (n = 4) equipped with these vibrators were placed back to their home cages and allowed another 7 days of habituation. After habituation, the rats were then exposed to two-week long SI regimen with a vibration cycle set to 10 seconds on and 290 seconds off, completing one cycle every five minutes throughout the 24-hour period (both light and dark phases) each day. Vibrators were recharged whenever the battery level dropped below 10%. The EEG/EMG signals were recorded on day 1, day 7 and day 14.

**Statistical analysis.** Parameters collected at baseline and various time points were analyzed using one-way repeated measures ANOVA, followed by Tukey’s test for pairwise comparisons. Data were presented as mean ± standard deviation (SD). All data were analyzed using SPSS 23.0 software. P values of < 0.05 were considered statistically signiﬁcant.

**Supplementary Results**

In the rats exposed to SI, on day 1 there was a distinct increase in the percentage of 24-h wake time, from 39.5 ± 4.3% at baseline to 59.7 ± 7.2% (P < 0.01). By day 7 and day 14, the percentage of 24-h wake time had declined to 52.4 ± 3.8% and 49.8 ± 4.5%, respectively, but the percentage on day 7 still had significant difference compared to baseline (P < 0.05). Whereas, no significant difference of wake time had been observed across time from day 1 to day 14 in the SI rats (Fig. 3B). In order to investigate whether SI fragmented sleep over the course of 2-week SI, the number of wake bouts and average sleep bout duration over 24 h were examined across the treatment time (Fig. 3C-D). The SI regimen led to an increased number of wake bouts on day 1, from 160.7 ± 23.88 at baseline to 259.0 ± 26.4 (P < 0.01); and a diminished sleep bout duration on day 1, from 3.85 ± 0.62 min at baseline to 1.51 ± 0.28 min (P < 0.001). No discernible alterations were observed in the number of wake bouts and the sleep bout duration across time from day 1 to day 14.

Supplementary Figure 1 Schematics of the automated sleep interruption system with remotely controlled vibrator.

Supplementary Figure 2 Automated sleep interruption system using remotely controlled vibrator. (A) Arrangement of the main control part consisted of electric power, programmable controller and wireless transmitter. (B) Front panel of the programmable control unit. (C) Front panel of the vibrating unit.

Supplementary Figure 3 Sleep architecture during SI procedure for 14 days. Data were collected on day 0 (baseline), day 1, day 7 and day 14. (A) Representative polysomnogramic (EEG and EMG) recordings scored as waking, NREM, and REM in rats. (B) Percentage of time in waking over 24 hours. (C) Number of wake bouts over 24 hours. (D) Average sleep bout duration over 24 hours. Data are expressed as mean ± SD. ***p < 0.001, **p < 0.01 and *p < 0.05 versus the baseline level. NREM, non-rapid eye movement sleep; REM, rapid eye movement sleep; SI, sleep interruption.
