# Supplementary material for: Neonatal supplementation of oleamide during suckling ameliorates maternal postpartum sleep interruption-induced neural impairment and endocannabinoid dysfunction in early adolescent offspring rats
Source: Front Nutr. 2025 May 12;12:1566182. doi: 10.3389/fnut.2025.1566182 (PMC12104719; doi:10.3389/fnut.2025.1566182)
Supplement: Supplementary file 2 [file Table_2.DOCX]

**Supplementary Table 1. Sequences of PCR primers**

| **Gene** | **Primer** |
| --- | --- |
| **Rat** |  |
| *β-actin* | Sense primer: 5′- ACGGTCAGGTCATCACTATCG -3′  Antisense primer: 5′- GGCATAGAGGTCTTTACGGATG -3′ |
| *Psd95* | Sense primer: 5′- ACTGCATCCTTGCGAAGCAAC -3′  Antisense primer: 5′- CGTCAATGACATGAAGCACATCC -3′ |
| *Syp* | Sense primer: 5′- AGGGCCTATGATGGACTTTCTG -3′  Antisense primer: 5′- TCCGTGGCCATCTTCACATC -3′ |
| *Il-1β* | Sense primer: 5′- CACCTCTCAAGCAGAGCACAG -3′  Antisense primer: 5′- GGGTTCCATGGTGAAGTCAAC -3′ |
| *Tnf-α* | Sense primer: 5′- AAATGGGCTCCCTCTCATCAGTTC -3′  Antisense primer: 5′- TCCGCTTGGTGGTTTGCTACGAC -3′ |
| *Il-10* | Sense primer: 5′- TTCCCTGGGAGAGAAGCTGA -3′  Antisense primer: 5′- ATGGCCTTGTAGACACCTTTGT -3′ |
| *Il-6* | Sense primer: 5′- TCCTACCCCAACTTCCAATGCTC -3′  Antisense primer: 5′- TTGGATGGTCTTGGTCCTTAGCC -3′ |
| **Mouse** |  |
| *β-actin* | Sense primer: 5′- CATTGCTGACAGGATGCAGAAGG -3′  Antisense primer: 5′- TGCTGGAAGGTGGACAGTGAGG -3′ |
| *Il-6* | Sense primer: 5′- TACCACTTCACAAGTCGGAGGC-3′  Antisense primer:5′- CTGCAAGTGCATCATCGTTGTTC-3′ |
| *Il-10* | Sense primer: 5′- CGGGAAGACAATAACTGCACCC -3′  Antisense primer: 5′- CGGTTAGCAGTATGTTGTCCAGC -3′ |
| *Tnf-α* | Sense primer: 5′- CATCTTCTCAAAATTCGAGTGACA-3′  Antisense primer: 5′- TGGGAGTAGACAAGGTACAACCC -3′ |

Il-1β, interleukin 1 beta; Il-6, interleukin 6; Il-10, interleukin 10; Tnf-α, tumor necrosis factor alpha; Psd95, postsynaptic density protein 95; Syp, synaptophysin.

**Supplementary Table 2.** Detailed two-way ANOVA results of the behavioral experiments.

| **Outcome Measure** | **Effects** | **Two-way ANOVA results** | |
| --- | --- | --- | --- |
| **Escape Latency in MWMT^a^** | Treatment | F (3, 21) = 5.825 | P = 0.0046 |
|  | Time | F (2.566, 53.90) = 37.70 | P < 0.0001 |
|  | Interactions | F (9, 63) = 1.614 | P = 0.131 |
| **Travel Distance in MWMT** | Treatment | F (3, 21) = 1.883 | P = 0.1635 |
|  | Time | F (2.879, 60.46) = 48.43 | P < 0.0001 |
|  | Interactions | F (9, 63) = 2.498 | P = 0.0164^b^ |
| **Swim Speed in MWMT** | Treatment | F (3, 21) = 0.711 | P = 0.556 |
|  | Time | F (2.494, 52.38) = 1.405 | P = 0.253 |
|  | Interactions | F (9, 63) = 0.588 | P = 0.801 |

^a^ MWMT, morris water maze test.

^b^ Because the interaction effect is significant between treatment and time, the overall P value is not meaningful. The post hoc analysis was performed among treatment groups at different time points.
