# Supplementary figures and images for: Neonatal supplementation of oleamide during suckling ameliorates maternal postpartum sleep interruption-induced neural impairment and endocannabinoid dysfunction in early adolescent offspring rats
Source: Front Nutr. 2025 May 12;12:1566182. doi: 10.3389/fnut.2025.1566182 (PMC12104719; doi:10.3389/fnut.2025.1566182)

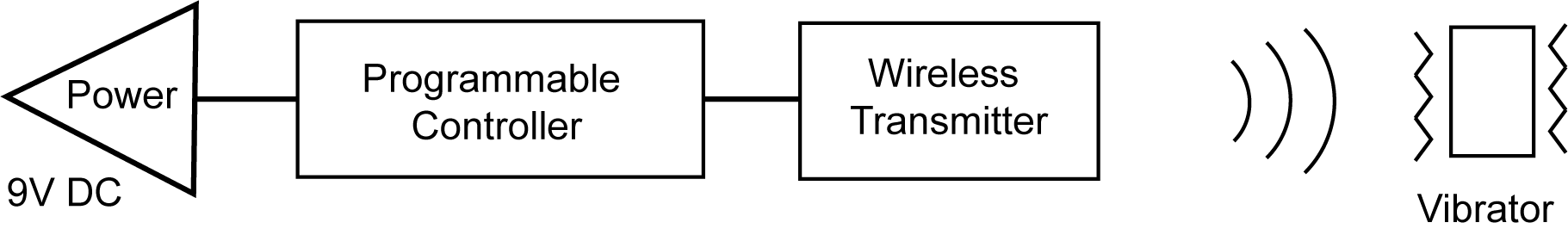

Supplement: Supplementary file 3 [file Image_1.TIF]

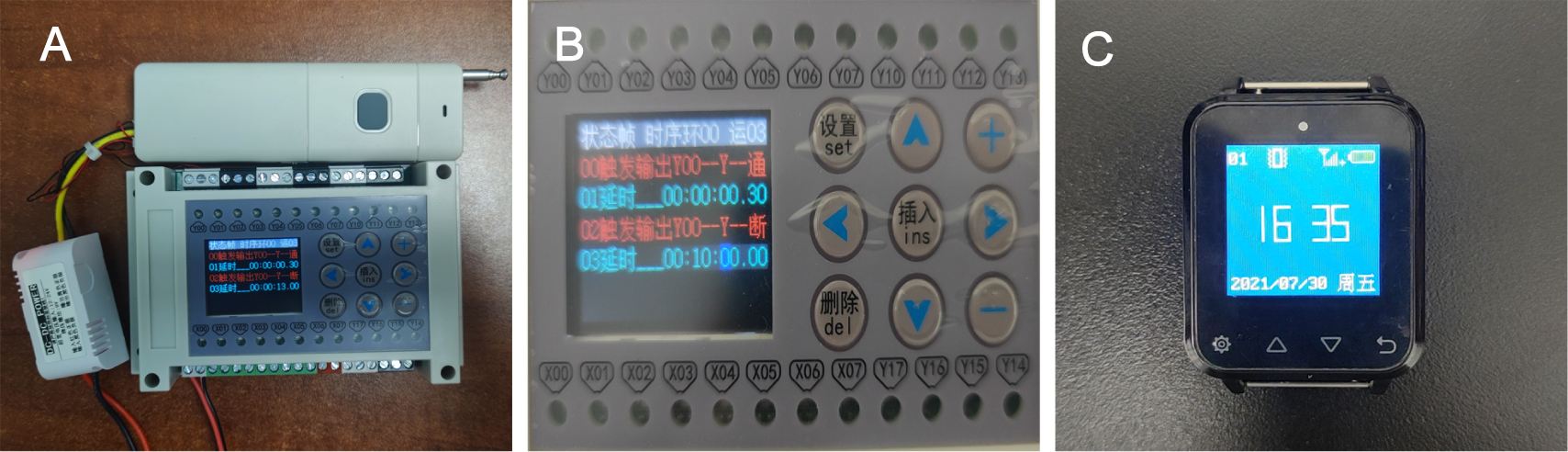

Supplement: Supplementary file 4 [file Image_2.TIF]

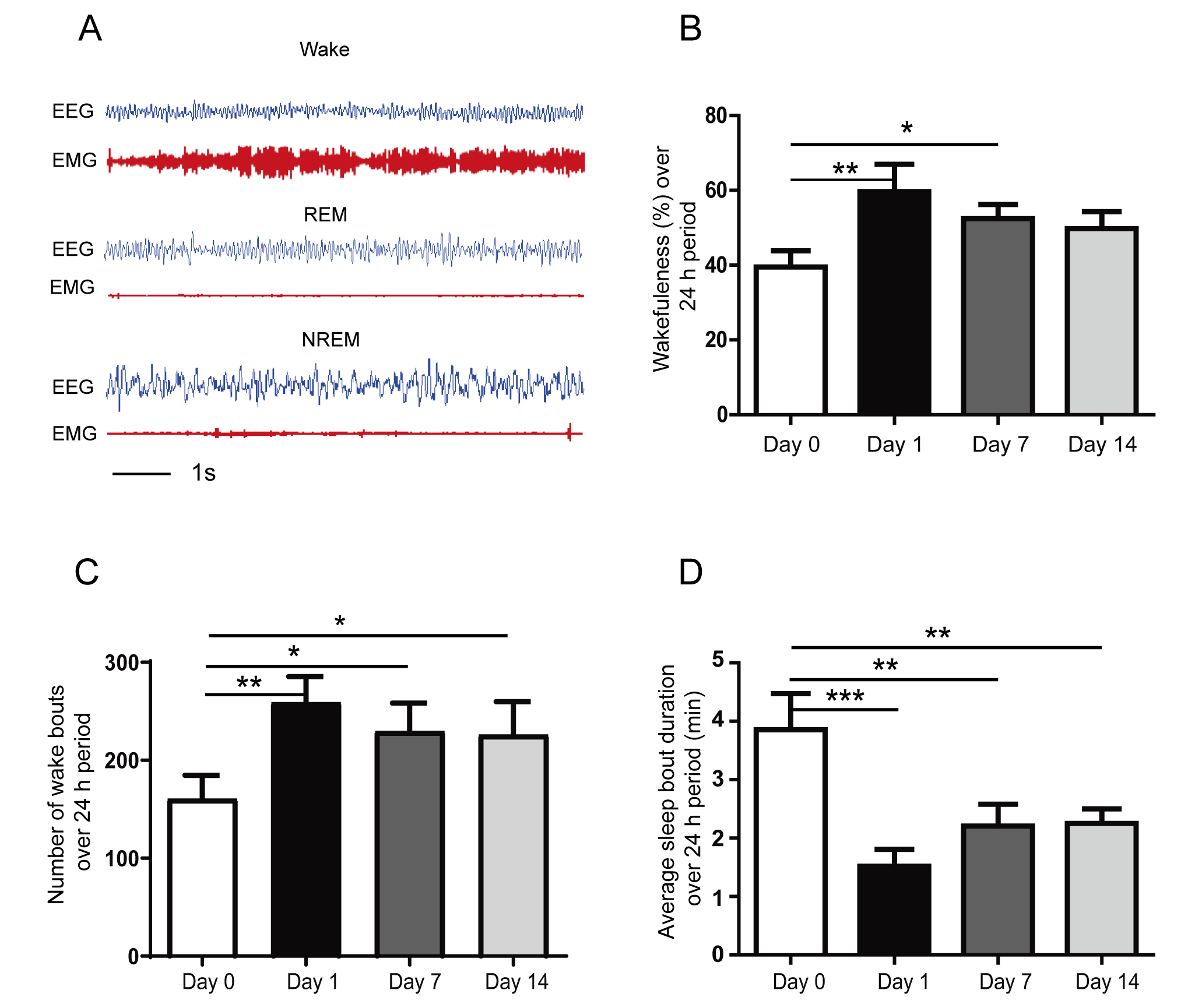

Supplement: Supplementary file 5 [file Image_3.TIF]
